# Hematopoietic stem/progenitor cell senescence is associated with altered expression profiles of cellular memory-involved gene

**DOI:** 10.1042/BSR20171589

**Published:** 2018-02-21

**Authors:** Yongpin Dong, Xiaolan Lian, Yanwu Xu, Haiyan Hu, Cen Chang, Haiyin Zhang, Lina Zhang

**Affiliations:** 1 Department of Biochemistry, Institute of Basic Medicine, Shanghai University of Traditional Chinese Medicine, Shanghai 201203, China; 2 Fujian Provincial Key Laboratory on Hematology, Fujian Institute of Hematology, Fujian Medical University Union Hospital, Fuzhou, China; 3 Department of Emergency and Critical Care Medicine, Shanghai Changzheng Hospital, The Second Military Medical University, Shanghai 200003, China

**Keywords:** cell senescence, cellular memory, hematopoietic stem/progenitor cells, β-galactosidase

## Abstract

To evaluate the contributions of cellular memory mechanisms to hematopoietic stem/progenitor cell (HSPC) senescence. HSPCs (Lin^−^CD117^+^, hereafter referred to as HSPC) were separated from young (6-week-old) and aged (18-month-old) mice using Magnetic Activated Cell Sorting (MACS). Cell cycle distribution of HSPCs was determined using flow cytometry. The mixed colony forming unit (CFU-Mix) assay was used to study the HSPCs’ ability to proliferate. The mRNA expression levels of cellular memory-implicated PCG family (enhancer of zeste homolog 2 (Ezh2), B lymphoma mo-MLV insertion region 1 (Bmi-1), embryonic ectoderm development (Eed), melanoma nuclear protein 18 (Mel18), Mph1/polyhomeotic-like protein 1 (Rae-28)) and Trithorax group (TrxG) family (mixed lineage leukemia (Mll), thioredoxin (Trx)) were determined by quantitative real-time PCR. We obtained highly purified populations of mouse HSPCs (Lin^−^CD117^+^) (92.2 ± 4.5% CD117^+^). The percentage of HSPCs was significantly higher in older mice compared with younger control mice and the percentage of SA-β-galactosidase positive cells was significantly higher in HSPCs isolated from older mice (*P*<0.05). The percentage of HSPCs in G_0_/G_1_ was significantly higher in older mice compared with younger control mice (52.0 compared with 47.1%), indicating increased cell cycle arrest in senescent HSPCs. The amount of CFU-Mix was significantly decreased in aged group (13.8 compared with 40.0), indicating a diminished ability to proliferate in senescent HSPCs. *Ezh1, Bmi-1, Eed, Rae-28* gene mRNA expression was significantly lower in HSPCs from older mice compared to younger controls, while *Mel18* mRNA expression was significantly higher in HSPCs from older mice (*P*<0.05). The expression of genes associated with cellular memory is altered in senescent (Lin^−^ CD117^+^) HSPCs, which may affect the potential plasticity of aged hematopoietic stem cells (HSCs) and thereby contribute to senescence-associated disease processes.

## Introduction

Senescence of hematopoietic stem/progenitor cells (HSPCs) is linked to aging, geriatric diseases, bone marrow hematopoietic depression, and leukemia [1]. Increased understanding of HSPC senescence mechanisms is important in defining aging and treating aging-associated diseases. To date, little attention has been focussed on cellular memory as a mechanism underlying hematopoietic stem cells (HSCs) senescence. Cellular memory is a process by which extracellular stimuli triggers permanent differentiation of stem cells into specific tissue types, associated with a regulated gene mRNA expression profile [2,3]. Cellular memory involves interactions between the members of the transcriptional repressor PcG (Polycomb group) and the transcriptional activator Trithorax group (TrxG) families. Interactions are maintained in a dynamic equilibrium [4] whereby *PcG* gene antagonizes *TrxG* gene-mediated transcriptional activation at the genomic level [5].

We used HSPCs isolated from 18-month-old mice as a model for cell senescence. We correlated cell senescence indicators with expression of PCG family important genes (B lymphoma mo-MLV insertion region 1, Bmi-1; enhancer of zeste homolog 2, Ezh2; embryonic ectoderm development, Eed; melanoma nuclear protein 18, Mel18; polyhomeotic-like protein 1, Rae-28) and TrxG family important genes (mixed lineage leukemia, Mll; thioredoxin, Trx) to evaluate cellular memory as a possible mechanism of HSC senescence. We found that cellular memory plays a role in HSPC senescence, which opens the window to identify potential therapies for aging-associated diseases.

## Materials and methods

### Animals

Equal numbers of male and female C57BL/6J specific pathogen free (SPF) mice were obtained from Shanghai Sippr-BK Experimental Animal Center (certificate number SCXK (Shanghai) 2013-0016). Young mice were 6 weeks of age and 18–22 g in weight, and old mice were 18 months of age and 25–30 g in weight.

### Reagents

Red blood cell lysis buffer, SA-β-Gal staining, and cell cycle kits were purchased from Beyotime Biotechnology Co., Ltd. Anti-c-kit (CD117) MicroBead, anti-stem cell antigen 1 (Sca-1) MicroBead, and Lineage Cell Depletion kits were purchased from Miltenyi Co., Ltd. MethoCult™ GF M3434 medium was purchased from Stem Cell Technologies Co. RNA Extraction and Purification, Reverse Transcription, and Fluorescence Quantitative PCR kits were purchased from Takara, Japan.

### Isolation and purification of HSPCs

Mice were killed by cervical dislocation. Bone marrow was removed from the femur and rinsed on to nylon mesh (30 μm pore size) under sterile conditions. The filtrate was centrifuged, the pellet was suspended in red blood cell lysis buffer, and then incubated at room temperature for 5 min. The lysate was centrifuged at 1400g for 5 min, the supernatant was discarded and the cell pellet was washed once. Bone marrow mononuclear cells (MNCs) were suspended in PBS containing 0.5 M EDTA and 0.5% BSA. HSPCs were obtained using anti-c-kit (CD117) microbeads and lineage cell depletion kits. The lineage cell depletion kit is a magnetic labeling system for the depletion of mature hematopoietic cells, such as T cells, B cells, monocytes/macrophages, granulocytes, and erythrocytes, and their committed precursors from bone marrow. All animal experiments were performed in compliance with the guidelines of the Animal Care and Use Committee of Shanghai University of Traditional Chinese Medicine.

### Flow cytometry

To test HSPC purification, 10^6^ MNCs (unpurified) and 10^6^ Lin^−^c-kit^+^ selected MNCs were collected and centrifuged at 400g for 5 min. The cells were washed once by PBS. Ten microliters of CD117-PE and Sca-1-FITC were added to the cells. The cells were incubated at 4°C for 15 min in the dark, washed once, resuspended in FACS buffer, and analyzed by flow cytometry using a Becton Dickinson AccuriTM C6.

### SA-β-gal (senescence-associated β-galactosidase) staining

HSPCs (10^6^) were fixed in 4% paraformaldehyde at room temperature for 15 min. The cells were washed with PBS and incubated at 37°C without CO_2_ for 16 h in β-galactosidase staining solution. The number of β-galactosidase positive cells per 400 total cells was counted under a microscope.

### Cell cycle testing

HSPCs (10^6^) were washed with cold PBS and fixed in cold 4% paraformaldehyde for 1 h at room temperature. Cells were centrifuged at 1000 ***g*** for 5 min, washed with PBS, and fixed overnight in 70% ethanol at 4°C. Cells were then centrifuged at 1000***g*** for 5 min, washed with PBS, and incubated in propidium iodide staining solution (Beyotime) at 37°C for 30 min in the dark. Flow cytometry was performed using an excitation wavelength of 488 nm. The cell cycle distribution was analyzed using FACS Express software.

### Mixed colony-forming unit of HSPC culture

Cells were diluted with IMDM + 2% FBS and MethoCult™ GF M3434 medium to a final concentration of 1 × 10^5^ per 35-mm dish. Diluted cells (0.3 ml) were thoroughly mixed with 3 ml of pre-aliquoted MethoCult™ in duplicate, and dispensed into 35-mm dishes with a total of 1.1 ml in each. The cells were incubated at 37°C, in 5% CO_2_ for 10 days. The percentage of mixed colony forming unit (CFU-Mix) per 5 × 10^3^ cells represented the pluripotency of the HSPCs.

### Quantitative RT-PCR

Total RNA extraction and reverse transcription were performed according to the manufacturer’s instructions for each kit used (9108/9109; RR047A; RR420A, Takara). The A260/280 ratio of RNA extracted from HSPCs was 1.8–1.9, indicating RNA of high purity. The primers used were:

β-actin (internal control): 5′-AACGCAGCTCAGTAACAGTCC-3′ (forward)
β-actin (internal control): 5′-GTACCACCATGTACCCAGGC-3′ (reverse)
Ezh2: 5′-AGCAGTAAGAGCAGCAGCAA-3′(forward)
Ezh2: 5′-TTCCTTCCATGCAACACCCA-3′ (reverse)
Bmi-1: 5′-GGACTGGGCAAACAGGAAGA-3′ (forward)
Bmi-1: 5′-GACTCTGGGAGTGACAAGGC-3′ (reverse)
Eed: 5′-GCTCAGCCTGATCGAATGCT-3′ (forward)
Eed: 5′-TTGGCGATGGGATCGACTTC-3′ (reverse)
Mel18: 5′-TCCCCATCTCCATTCTCCGT-3′ (forward)
Mel18: 5′-ATACCCCCTGACAGAGGTCC-3′ (reverse)
Rae-28: 5′-GCACAGATCTTGAGAGCAGG-3′ (forward)
Rae-28: 5′-GCAAGGCTGCCAAGAGATTG-3′ (reverse)
Trx: 5′-TAAAGCAGTGGCTTAGGGGAC-3′ (forward)
Trx: 5′-GAGAGTCTATACCCAACTGCCA-3′ (reverse)
Mll: 5′-ACGCTTGTCTGTCTGGATGG-3′ (forward)
Mll: 5′-CCCATGAGATTCCGGCACTT-3′ (reverse).

YBR green dye was used for real-time PCR. The 2^−Δ*C*^_t_ method was used to calculate mRNA expression levels. Δ*C*_t_ = *C*_t target gene_ – *C*_t internal control gene_ (where *C*_t_ is the cycle number when the fluorescence signal reaches the set threshold). The amplification parameters were: 95°C for 30 s (95°C for 5 s, 60°C for 34 s) for 40 cycles.

### Statistical analysis

The data were expressed as the mean ± S.D. Single-factor ANOVA was performed using SPSS 18.0. The LSD or Tamhane test was used to compare difference between the two groups. *P*<0.05 was considered statistically significant.

## Results

### Purification of mouse HSPCs

**Figure 1:**
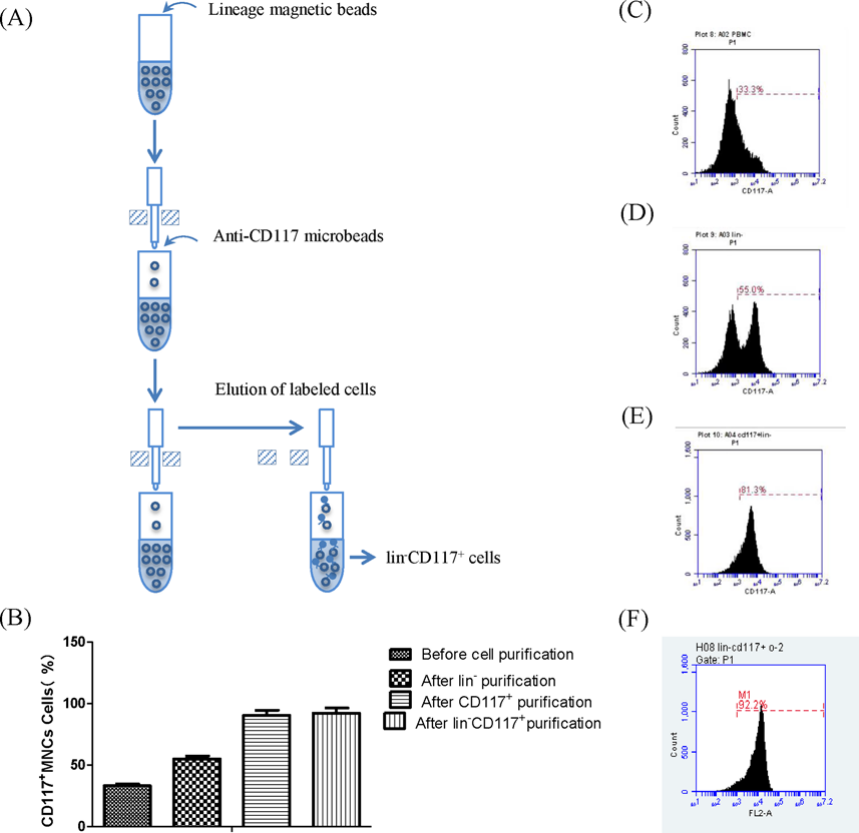
HSPCs that were effectively purified by using MACS and HSPCs purification were assessed using flow cytometry (**A**) For lineage depletion, cells are magnetically labeled with a cocktail of biotinylated antibodies against a panel of so-called ‘lineage’ antigens (CD5, CD45R (B220), CD11b, anti-Gr-1 (Ly-6G/C), 7-4, and Ter-119 antibodies) and anti-biotin microbeads. This labeling procedure leaves lineage negative cells untouched, thus allowing further separation of lineage^–^ cells according to expression of markers such as CD117. And for positive selection, anti-CD117/c-kit microbead kit was used. (**B**) Flow-cytometric analysis of CD117+ populations in mice MNCs in different phases of purification. Graphs show the population of CD117^+^ in MNCs (**C**) before cell purification, (**D**) after lineage depletion, (**E**) after CD117-positive selection, and (**F**) after Lin^−^c-kit^+^purification.

**Figure 2:**
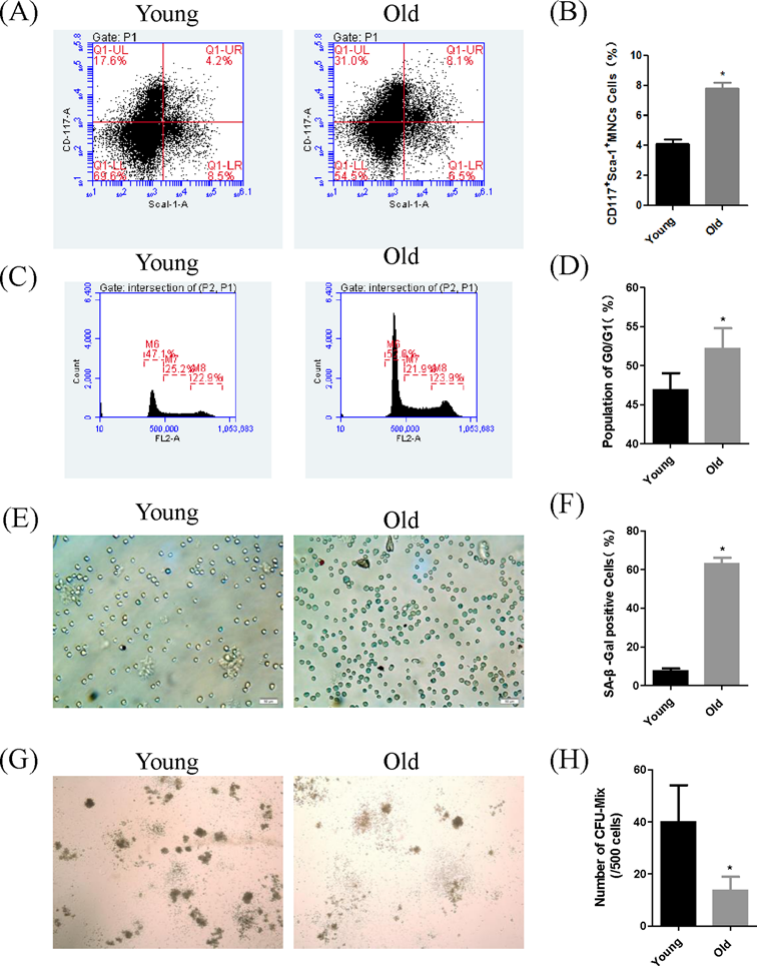
HSPCs in older mice show cell senescence, had G_0_/G_1_ arrest and decreased stem cell repopulating activity (**A**) CD117^+^Sca-1^+^cells in MNCs were identified by flow cytometer. (**B**) Graph shows the percentage of CD117^+^ Sca-1^+^ MNCs isolated from young and old mice; *n*=4. (**C**) Flow-cytometric analysis of cell cycle in the mice HSPCs show that senescent HSPCs were arrested in G_0_/G_1_. (**D**) Graph shows the population of G_0_/G_1_ cell cycle distribution of HSPCs in young and old mice; *n*=4. (**E**) Photomicrographs show SA-β-Gal staining in HSPCs isolated from young and old mice (×200). (**F**) Graph shows the percentage of SA-β-Gal positive HSPCs were significantly increased in aged mice, *n*=10. (**G**,**H**) HSPCs from young and old mice were incubated in MethoCult™ M3434 (5 × 10^3^ per 35-mm dish) medium at 37°C, in 5% CO_2_ for 10 days. The sizes and numbers of CFU-Mix of HSPCs were significantly decreased in aged mice, *n*=4.The results are expressed as mean ± S.D. and the *P* values (*P<0.05) determined by Student’s *t* test.

HSPC purity was assessed using flow cytometry. The percentage of CD117^+^ MNCs before selecting was 33.2 ± 1.4% (*n*=4). After Magnetic Activated Cell Sorting (MACS), Lin^−^ selecting the purity of CD117 ^+^ cells was 55.0 ± 2.4% (*n*=4). After MACS CD117^+^ selecting, CD117^+^ cell purity was 81.3 ± 4.1% (*n*=4). MACS Lin^−^CD117^+^ selecting yielded CD117^+^ cell purity of 92.2 ± 4.5% (*n*=10) (Figure 1). This indicates that HSPCs selected by the Lin^−^c-Kit/C**D**117^+^ are highly purified and suitable for subsequent experiments. Furthermore, the proportion of CD117^+^Sca-1^+^ cells was significantly higher in MNCs from older mice compared with younger control mice (Figure 2A,B), which indicates an age-related increase in HSPCs.

### SA-β-gal positive HSPCs were significantly increased in aged mice

SA-β-gal is a cell senescence marker yielding a blue stain in the cytoplasm of aging cells. We observed a higher percentage of SA-β-galactosidase positive HSPCs in older mice compared with younger controls (Figure 2E,F and Table 1).

**Table 1:** Percentage of SA-β-Gal positive HSPCs. (% $\bar{x}$ ± s, *n*=10)

Group	*n*	SA-β-Gal positive cells (%)
Young	10	7.78 ± 1.04
Old	10	63.08 ± 2.98*

* *P*<0.05, compared with the control group.

### Senescent HSPCs were arrested in G_0_/G_1_

The G_0_/G_1_ rate in senescent HSPCs (52.2%) was higher than younger mice (46.9%), while the percentage of S-phase cells in senescent HSPCs (21.6%) was decreased relative to younger mice (25.9%) (Figure 2C,D). These results suggest a greater degree of cell cycle arrest in senescent HSPCs from older mice relative to younger control.

### The percentage of CFU-Mix per 5 × 10^3^cells was significantly decreased in aged HSPC

There was a significant decrease in the percentage of CFU-Mix formed by 5 × 10^3^ HSPCs from old mice compared with the young control (Figure 2G,H and Table 2). CFU-Mix represented the multidifferentiation potential of the HSPCs, and the CFU formation ability is one of the major indexes for evaluating HSPC aging and functions. When HSPCs age, the capacity to form CFU-Mix colonies reduces with diminished self-renewal and multidifferentiation potential. From our results, we also can easily find a negative correlation existing between the positive rate of SA-gal and the CFU-Mix.

**Table 2:** Percentage of CFU-Mix of HSPCs (% $\bar{x}$ ± s, *n*=4)

Group	*n*	Percentage of CFU-Mix (per 500 cells)
Young	4	9.72 ± 10.41%
Old	4	4.10 ± 5.1%*

* *P*<0.05, compared with the control group

### The mRNA expression profiles of *PcG*, but not *TrxG* genes change with HSC aging

**Figure 3:**
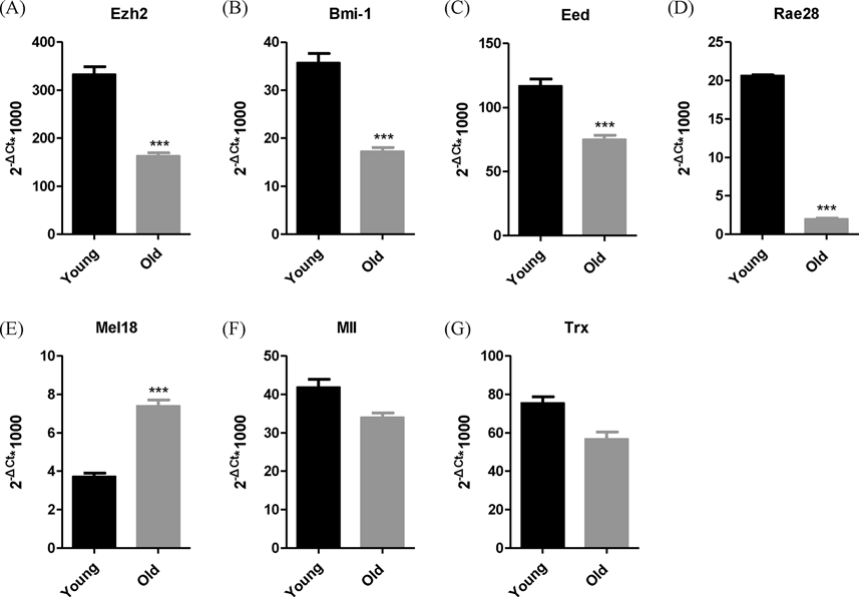
The mRNA expression profiles of *PcG* genes change with HSPCs aging Real-time quantitative PCR was used to detect the relative transcription levels of PcG family members and Trx family members; *n*=4.The transcription levels of Ezh1 (**A**), Bmi-1 (**B**), Eed (**C**), and Rae-28 (**D**) were significantly lower in older HSPCs compared with younger HSPCs. *Mel18* (**E**) mRNA levels, by contrast, were higher in older HSPCs. The HSPCs expression levels of *Mll* (**F**) and *Trx* (**G**) genes were not significantly different in older compared with younger HSPCs; 2^−Δ*C*^_t_ method was used to calculate mRNA expression levels. The results are expressed as mean ± S.D. and the *P* values (*P<0.05, **P<0.01, ***P<0.001) determined by Student’s *t* test.

The transcription levels of Ezh1, Eed, Bmi-1, and Rae-28 were significantly lower in aged HSPCs compared with young HSPCs. *Mel18* mRNA levels, by contrast, was higher in aged HSPCs. The transcription levels of *Mll* and *Trx* genes were not significantly different in aged compared with young HSPCs (Figure 3 and Table 3).

**Table 3:** PcG family members gene expression of HSPCs/β-actin ($\bar{x}$ ± s, *n*=4)

Group	Ezh1	Bmi-1	Eed	Mel18	Rae-28	Mll	Trx
Young	332.62 ± 15.98	35.70 ± 1.92	116.78 ± 5.49	3.73 ± 0.18	20.64 ± 0.09	41.87 ± 2.04	75.46 ± 3.29
Old	162.89 ± 6.38***	17.24 ± 0.82***	74.94 ± 3.36***	7.40 ± 0.31*	1.99 ± 0.13**	34.01 ± 1.14	56.79 ± 3.58

* *P*<0.05, compared with the control group.

## Discussion

Currently, the mechanism of HSC senescence is poorly understood owing in part to the low numbers of karyotypes in the bone marrow. Here positive and negative MACS systems can be used to isolate and purify mouse Lin^−^c-Kit^+^ cells, namely HSPC, at >85% purity [6,7]. HSPCs isolated from 18-month-old mice were used as a model for cell senescence. In this model, the percentage of SA-β-gal positive cells and the ratio of G_0_/G_1_ cells were both significantly increased relative to HSPCs purified from younger control mice, indicating stem cell senescence. The previous studies showed that HSCs in older mice had decreased potential for per cell repopulating activity and self-renewal [8]. In our research, we also found that the significant reduction in the number of CFU mixtures formed by old group HSCs, indicated stem cell senescence. We also found that significantly more HSCs were associated with aging mice consistent with previous observations that numbers of HSCs increase with age [7,9].

Studies on HSC senescence have largely focussed on telomeres, DNA damage, and oxidative injury with little attention to cellular memory [10]. Cellular memory involves a dynamic equilibrium between PcG and Trx family proteins. PcG proteins repress transcription of target genes through chromatin modification, and act as novel epigenetic factors in stem cell fate determination. PcG consists mainly of two protein complexes: polycomb repressive complex 1 (PRC1) and polycomb repressive complex 2 (PRC2) [11–13]. PRC1 comprises Rae-28, Bmi-1, Mel18, and other proteins that maintain the stability of a repressive chromatin state [14]. PRC2 comprises Eed, Ezh2, and other proteins [15] that can methylate histones at HlK26 and H3K27 and thereby repress target gene transcription [16]. Ezh2 is a key member of the PcG family. It can promote the survival of HSCs [17,18]. Ezh2 forms a complex with Eed that silences target genes. We found that the expression of *Ezh2* and *Eed* genes was significantly reduced in senescent HSPCs relative to yong HSPCs thus, suggesting that Ezh2 and Eed might delay HSPC senescence. PRC1 complex member Bmi-1 regulates cell proliferation, senescence, and immortalization through multiple distinct pathways. Mutations of the *Bmi-1* gene results in decreased numbers of bone marrow-derived hematopoietic progenitor cells and reduced cell proliferation [19]. In this study, Bmi-1 expression in senescent HSPCs was significantly lower than in young HSPCs, again suggesting a role for Bmi-1 in delaying HSPC senescence. Mel18 negatively regulates the self-renewal capacity of HSPCs, and *Mel18* gene silencing leads to increased numbers of HSPCs in G_0_ phase of the cell cycle. We also found that Mel18 transcript level was significantly higher in senescent HSPCs compared with younger HSPCs. The previous studies show that Rae-28 had a crucial role in sustaining the activity of HSCs to maintain hematopoiesis [20]. Our study showed that Rae-28 expression in senescent HSPCs was significantly lower than in younger HSPCs. In contrast with PcG proteins, Trx-mediated histone modification promotes target genes transcription through chromatin modification. In TrxG protein family, the key members are SET domain-containing proteins, including Trx, Ashl, and Mll [21]. Previous studies show that Mll modulates myeloid cell differentiation by regulating *HOXA10* gene expression in acute myeloid leukemia cells [22]. Whereas Trx promotes cell survival, cell proliferation, and responses to oxidative stress [23]. However, we found no differences in *Mll* and *Trx* mRNA levels of senescent and young HSPCs, suggesting that Trx group member dynamics do not contribute to cellular memory dysregulation in HSPC senescence in mouse.

Under physiological conditions, the PcG and TrxG family proteins can maintain cellular memory and prevent alteration of HSCs identity by promoting or repressing the expression of target genes, respectively. Our findings suggest that PcG family gene mRNA expression changes as HSPCs age, whereas *TrxG* gene mRNA expression does not. Therefore we conclude that altered HSPC cellular memory, leading to cell senescence and age-related diseases, results from an abrogation of PcG-TrxG regulatory equilibrium in HSPCs of aging mice. These observations extend our understanding of the molecular mechanisms underlying HSC senescence and aging-associated diseases.

## Abbreviations

Bmi-1: B lymphoma mo-MLV insertion region 1
CFU-Mix: mixed colony forming unit
Eed: embryonic ectoderm development
Ezh2: enhancer of zeste homolog 2
HSC: hematopoietic stem cell
HSPC: hematopoietic stem/progenitor cell
MACS: magnetic activated cell sorting
Mel18: melanoma nuclear protein 18
Mll: mixed lineage leukemia
MNC: bone marrow mononuclear cell
PcG: polycomb group
PRC1: polycomb repressive complex 1
PRC2: polycomb repressive complex 2
Rae-28: polyhomeotic-like protein 1
Sca-1: stem cell antigen 1
Trx: thioredoxin
TrxG: trithorax group
SA-β-gal: senescence-associated β-galactosidase
